# Adverse Childhood Experiences and Mental Health Among Adolescents, Young Adults, and Adults: Mediating Roles of Positive Childhood Experiences and Resilience

**DOI:** 10.1007/s11126-026-10286-3

**Published:** 2026-04-30

**Authors:** İlhan Çiçek, Murat Yıldırım

**Affiliations:** 1 Health College, Batman University, Batman, Türkiye; 2 Department of Psychology, Faculty of Science and Letters, Ağrı İbrahim Çeçen University, Fırat Mahallesi Yeni Üniversite Caddesi No: 2 AE/1, Merkez, Ağrı, 04100 Türkiye; 3 Psychology Research Center, Khazar University, Baku, Azerbaijan

**Keywords:** Positive childhood experiences, Resilience, Adverse childhood experiences, Anxiety, Depression

## Abstract

Adverse childhood experiences (ACEs) are well-established risk factors for poor mental health, yet less is known about how positive childhood experiences (PCEs) and resilience may buffer these effects across different stages of life. Existing research has primarily focused on ACEs in isolation, leaving a gap in understanding the protective mechanisms that promote psychological well-being despite adversity. To address this gap, the present study examines the effects of ACEs and the mediating roles of PCEs and resilience in their relationship with anxiety and depression. The sample consisted of 2,287 Turkish participants, including adolescents (ages 13–18, *n* = 683), young adults (ages 19–29, *n* = 1,076), and adults (ages 30–75, *n* = 528). Participants completed the Adverse Childhood Experiences (ACEs) scale, the Positive Childhood Experiences (PCEs) scale, the Resilience Scale, and the Depression, Anxiety, and Stress Scale-21 (DASS-21). Results showed that ACEs were negatively associated with PCEs and resilience across all groups, while they were positively linked to anxiety and depression. Mediation analyses revealed that PCEs and resilience significantly mediated the relationship between ACEs and mental health outcomes. These findings underscore the importance of fostering positive childhood experiences and resilience as protective factors that can mitigate the long-term psychological impact of ACEs, informing targeted prevention and intervention strategies to support mental health across different life stages.

## Introduction

Childhood experiences significantly influence an individual’s lifelong development, affecting emotional, cognitive, and social well-being. Both positive and negative experiences have lasting impacts on well-being and mental health. Research highlights how adverse childhood events can lead to long-term psychological challenges, while positive experiences can foster resilience and better mental health outcomes [1]. Understanding these influences is important for developing effective interventions across different age groups.

Adverse childhood experiences (ACEs) include traumatic events experienced by individuals before the age of 18 [2]. ACEs represent exposure to subversive situations such as physical, emotional, and sexual abuse, bullying, and growing up in unsafe home environments. Furthermore, ACEs strongly predict physical and mental health [3–6]. ACEs are a major issue with shattering effects on individuals and societies and a significant public health concern [7]. Many studies have indicated that ACEs are associated with anxiety, depression, hostility, loneliness, and other health problems [8–12]. Additionally, individuals with a history of ACE hold a higher risk of experiencing a range of adverse mental and physical health problems throughout their lives [3–5, 13]. In the existing literature, on one hand, ACEs are negatively associated with PCEs [14], resilience [15], empathy [16], self-esteem [17], and mental well-being [18, 19]. On the other hand, ACEs increase anxiety and depression and have greater detrimental effects [14, 20, 21]. The greater the exposure to ACEs during adolescence, the higher the likelihood of experiencing anxiety, depression, and poor mental health outcomes later in life [11, 22]. Similarly, research suggests that ACEs increase the severity of anxiety, depression, and post-traumatic stress disorder [23]. All this strong evidence revealed that ACEs have multiple devastating effects across the lifespan.

Following the discussion of ACEs, it is important to highlight their established associations with common mental health outcomes, particularly the prevalence of depression and anxiety. Anxiety and depression, defined as emotional disorders characterised by excessive feelings of worry, fear, sadness, or hopelessness, have destructive effects on individuals at every stage of life [24]. Anxiety and depression are increasingly recognised as two global public health problems [25]. Further, anxiety and depressive disorders are common and debilitating mental disorders that affect approximately 10% of the world’s population each year (WHO, [26]). Previous studies demonstrated that high levels of anxiety and depression are negatively associated with positive variables such as quality of life [27], PCEs (Şanlı et al., 2024), and resilience [28]. Additionally, previous studies have documented that individuals exposed to ACEs are more likely to experience higher levels of anxiety and depression in adolescence [22, 29], young adulthood [30], and adulthood [45]. Therefore, this makes individuals more vulnerable to adversity [1, 10] and causes deep personal suffering [31]. In this context, it is emphasized that anxiety and depression negatively impact individuals’ lives and lead to further mental health problems (Çeri & Çiçek, [32]; [5]).

## Mediation Role of Positive Childhood Experiences (PCEs) and Resilience

PCEs are defined as positive/advantaged childhood experiences before the age of 18, including warm, nurturing, and supportive care, a stable family environment, safe and equitable environments, and active participation in school [1, 21, 33, 34]. Individuals exposed to many PCEs tend to lead a happy, healthy, and productive life throughout adulthood ([1]; Çiçek & Çeri, [35]). In particular, PCEs are exploited as a buffer against the negative effects of ACEs. Furthermore, PCEs have been reported to act as a significant stabilizer of ACEs [1, 14, 36], diminish the impacts of ACEs [21, 37, 38], and hold an empowering and developing role on individuals [1, 39–42]. Additionally, PCEs’ abundance has been reported to mitigate negative mental health problems such as anxiety and depression [34, 43]. Another interesting finding is that when the number of ACEs is low, PCEs can offset the adverse effects of ACEs on adult health [21, 38]. Being exposed to PCEs in large amounts brings about lower depression, anxiety, and stress and more positive body image, self-esteem, life satisfaction, and well-being [14, 18, 39, 41, 44]. Notably, the effects of PCEs on mental health have been reported to be more positive in the presence of low ACEs [45]. Systematic review studies provided evidence for the potential protective effects of PCEs against ACEs [43] and suggest that PCEs may moderate the impact of ACEs and play a key role in alleviating the risk of mental disorders and other adverse outcomes later in life [6]. All these empirical study results indicate a significant protective and developmental potential of PCEs.

The concept of resilience is defined as the individual’s capacity to bounce back and recover, adapt to the environment, and endure difficulties, and it is influenced by factors such as supportive relationships, emotional regulation, and problem-solving skills [46–48]. Bonanno, [49] interpreted resilience as an all-purpose characteristic that facilitates an individual’s psychological well-being after traumatic events [50]. Resilience, a functional personality trait, has been reported to minimize the devastating effects of traumatic/negative events [51], diminish depression and anxiety levels [52], serve as a safeguard against traumatic events [28, 53], and protect against psychopathology in the regard of trauma [54]. Remarkably, resilience enables individuals to be strong in navigating challenging conditions and maintaining positive relationships [55, 56] and plays a core role in reducing the devastating effects of ACEs [57, 58].

### Theoretical Framework

The study is grounded in the Developmental Psychopathology framework [59], which comprehensively addresses how risk and protective factors interact across developmental stages and shape psychosocial outcomes. Within this framework, ACEs, including abuse, neglect, and family dysfunction, are conceptualized as risk factors that negatively impact mental health. In contrast, PCEs and resilience are considered protective mechanisms that support psychological well-being and can mitigate the adverse effects of ACEs [1, 60]. Accordingly, the proposed mediation model in this study is theoretically well-founded: ACEs are expected to exert direct negative effects on mental health outcomes, while PCEs and resilience play a mediating role in the indirect pathways through which these effects manifest. The study aims to examine the impact of ACEs on mental health across different age groups (adolescents, young adults, and adults) and to explore the mediating role of PCEs and resilience, thereby providing a theoretically informed and comprehensive contribution to the existing literature.

## Present Study

**Fig. 1:**
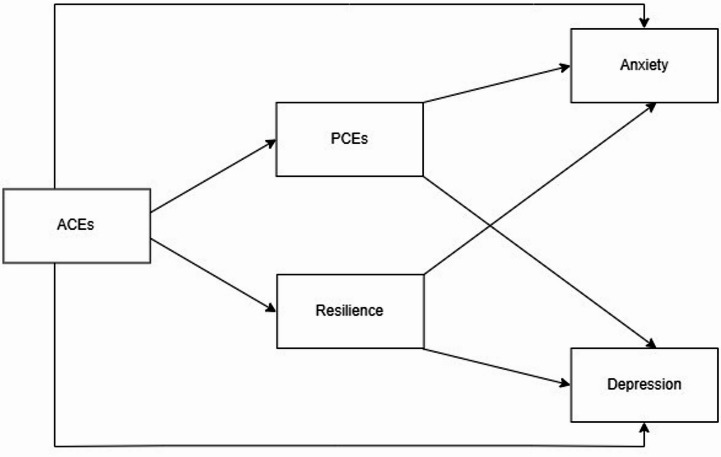
The proposed model of relationships between variables

This study aimed to examine whether PCEs and resilience mediate the relationship between ACEs and anxiety and depression across adolescent, young adult, and adult samples. While extensive research has highlighted the detrimental effects of ACEs [7, 61–63], recent studies have increasingly explored the protective role of PCEs [41, 42, 64, 65]. However, no research to date has investigated the mediating roles of PCEs and resilience in this relationship, and limited attention has been given to how these factors may explain the association of ACEs with anxiety and depression across different developmental stages. Moreover, important questions remain regarding how these mediating factors operate among adolescents, young adults, and adults. By addressing these gaps, the present study contributes to a more nuanced understanding of the relationships between ACEs, PCEs, resilience, and mental health outcomes across the lifespan. Additionally, this study is unique in its simultaneous examination of these factors across different life stages, providing a broader perspective on their long-term impact on mental health. The hypotheses of the study are as follows: (H_1_) ACEs will be negatively associated with PCEs and resilience, and will be positively associated with anxiety and depression; (H_2_) PCEs and resilience will be negatively associated with anxiety and depression; (H_3_) PCEs and resilience will serve as mediators in the association between ACEs and anxiety and depression. The hypothesized model is presented in Fig. 1.

## Method

### Participants and Procedures

The current study was designed using a convenience sampling method. The data were collected online from adolescents, young adults, and adults via social media platforms (WhatsApp, Instagram, etc.) between November 18, 2024, and January 6, 2025, in Türkiye. Before the data collection process began, ethics committee approval was obtained from Batman University (Ethics Code: 2024/11–36). Consent forms were obtained from the parents of participants under the age of 18 through school principals. In addition, participants were informed about the purpose of the study, confidentiality of personal information, and voluntary participation, and were assured of their rights. Written informed consent was obtained from all participants included in the study.

The study sample comprised 2,287 participants, including adolescents, young adults, and adults from various regions of Türkiye. Of these, 1,355 were female (59.2%), and 932 were male (40.8%), with ages ranging from 13 to 75. The adolescent group included 683 participants (Mage = 15.95, SD = 1.33), consisting of 432 females and 252 males, aged between 13 and 18. The young adult group comprised 1,076 participants (Mage = 21.94, SD = 2.43), including 692 females and 384 males, aged between 19 and 29. The adult group consisted of 528 participants (Mage = 38.69, SD = 7.75), with 230 females and 298 males, aged between 30 and 75. The majority of the adult participants (65%) were married.

### Measures

#### Adverse Childhood Experiences (ACEs)

ACEs consist of 10 items related to adverse events experienced by individuals before the age of 18. The items measuring ACEs have been defined and standardized by the Centers for Disease Control and Prevention (CDC). The validity and reliability study of the scale was conducted by Felitti et al. (1998). Participants answer the scale questions with “yes” or “no”. Scores on the scale range from 0 to 10, with higher scores indicating that individuals have more ACEs. A sample item is “Has anyone in your household been depressed or mentally ill, or attempted suicide?” The Turkish adaptation of ACEs was made by Gündüz et al. (2018). In the current study, Cronbach’s alpha value for ACEs was 0.86 for the adolescent group, 0.88 for the young adult group, and 0.89 for the adult group.

#### Positive Childhood Experiences (PCEs)

PCEs, developed by Bethell et al., [1], measure individuals’ positive experiences before the age of 18. The scale consists of 7 items and uses a 5-point Likert scale, ranging from 1 (Never) to 5 (Always). The highest score that can be obtained from PCEs is 35, and the lowest score is 7. A sample item is “How much of your childhood was there an adult who made you feel safe and protected at home?” The Turkish adaptation of the scale was made by Çiçek and Çeri [35]. In the current study, Cronbach’s alpha value for PCEs was found to be 0.75 for the adolescent group, 0.78 for young adults, and 0.76 for the adult group.

#### Resilience Scale (RS)

The scale developed by Merino and Privado [66] is a 3-item, unidimensional scale that assesses the capacity to cope with difficulties and maintain resilience. The scale is a 5-point Likert-type scale. An example item is “I get stronger as I face challenges.” The Turkish adaptation of the scale was made by Green et al., [67]. In the current study, Cronbach’s alpha value for RS was found to be 0.76 for the adolescent group, 0.79 for the young adult group, and 0.83 for the adult group.

#### Depression, Anxiety, Stress Scale (DASS-21)

DASS-21, developed by Lovibond and Lovibond [68], consists of 21 items and 3 subscales: Depression, Anxiety, and Stress. Each subscale consists of 7 items and has a 4-point Likert-type rating. The validity and reliability study of the short form of the scale was conducted by Henry and Crawford [69]. A minimum of 0 and a maximum of 21 points can be obtained from each subscale. High scores on the scale indicate high levels of depression, anxiety, and stress. In the current study, depression and anxiety subscales were used. A sample item for anxiety is “I tend to overreact to events”. A sample item for depression is “I felt that life was worthless”. The Turkish adaptation of DASS-21 was conducted by Yılmaz et al., [70]. In the study, Cronbach’s alpha values were 0.87 and 0.73 for anxiety of the adolescent group; 0.86 and 0.77 for depression of young adults; and for the adult group, anxiety was 0.89 and depression was 0.75.

### Data Analysis

In the current study, data analyses were conducted in three stages. In the first stage, descriptive statistics, including means, standard deviations, and percentages, were calculated to summarize the data. In the second stage, bivariate correlations were computed to examine the associations among the relevant variables. In the third stage, mediation analyses were performed using PROCESS macro Model 4, with 5,000 bootstrap samples to estimate the 95% confidence interval of the indirect effects [71, 72].

## Results

### Preliminary Analysis

Before passing to the main analysis, the relationship between the variables in the study, the values ​​of the variables, the standard deviation, averages, and Cronbach’s alpha values ​​of the variables were examined (See Table 1). Analyses indicate that the kurtosis (between 2.67 and − 0.66) and skewness values ​​(between + 1.67 and − 0.57) of all three study groups ​​were within acceptable limits, indicating a normal distribution [73]. Pearson’s Moment Correlation was used to examine the relationship between variables. Analyses show that there is a negative and significant association between ACEs and PCEs and resilience in all three groups, and a positive and significant association between ACEs and anxiety and depression. Additionally, a negative and significant relationship was found between PCEs and resilience, as well as anxiety and depression. ACEs are positively associated with anxiety and depression, this result indicating that exposure to adverse experiences during childhood is likely to contribute to the development of anxiety and depressive symptoms later in life. Conversely, ACEs are negatively associated with both PCEs and resilience. This situation suggests that a higher number of adverse experiences in childhood is linked to a reduction in PCEs and a decrease in resilience. Cronbach’s alpha values ​​of the measurement tools used in the study ranged between (α)=0.73 and (α)=0.89 in all three groups for all scales (see Table 1). This result indicates that the scales are reliable for this study.

**Table 1:** Correlation coefficient, internal consistency reliability, and descriptive statistics among the study variables

	Correlations													Reliability	Descriptive statistics			
Variable	1.			2.			3.			4.			5.	α	Mean	SD	Skewness	Kurtosis
Adolescents																		
1. ACEs	—													0.86	1.19	1.70	1.67	2.57
2. PCEs	−0.37^**^			—										0.75	23.91	5.54	−0.44	−0.05
3. Resilience	−0.15^**^			0.35^**^			—							0.76	10.56	2.89	−0.49	−0.01
4. Anxiety	0.35^**^			−0.29^**^			−0.23^**^			—				0.87	7.43	5.80	0.55	−0.67
5. Depression	0.42^**^			−0.47^**^			−0.26^**^			0.59^**^			—	0.73	7.63	5.50	0.51	−0.56
	Correlations														Descriptive statistics			
Young adults	1.			2.			3.			4.			5.		Mean	SD	Skewness	Kurtosis
1. ACEs	—													0.88	1.39	1.94	1.66	2.68
2. PCEs	−0.44^**^			—										0.78	23.99	5.50	−0.40	0.14
3. Resilience	−0.13^**^			0.30^**^			—							0.79	10.99	2.63	−0.66	0.52
4. Anxiety	0.28^**^			−0.23^**^			−0.18^**^			—				0.86	6.65	5.31	0.62	−0.35
5. Depression	0.29^**^			−0.31^**^			−0.24^**^			0.58^**^			—	0.77	6.94	5.27	0.70	−0.12
5. Depression	0.29^**^			−0.31^**^			−0.24^**^			0.58^**^			—	0.77	6.94	5.27	0.70	−0.12
	Correlations														Descriptive statistics			
Adults	1.			2.			3.			4.			5.		Mean	SD	Skewness	Kurtosis
1. ACEs	—													0.89	1.55	1.96	1.37	1.52
2. PCEs	−0.46^**^			—										0.76	24.41	5.20	−0.58	0.28
3. Resilience	−0.19^**^			0.24^**^			—							0.83	11.29	2.68	−1.02	1.18
4. Anxiety	0.30^**^			−0.25^**^			−0.26^**^			—				0.89	4.11	4.60	1.40	1.65
5. Depression	0.30^**^			−0.35^**^			−0.32^**^			0.63^**^			—	0.75	5.14	4.86	1.22	1.27

**. Correlation is significant at the 0.01 level (2-tailed)

### Parallel Mediation Analysis

**Fig. 2:**
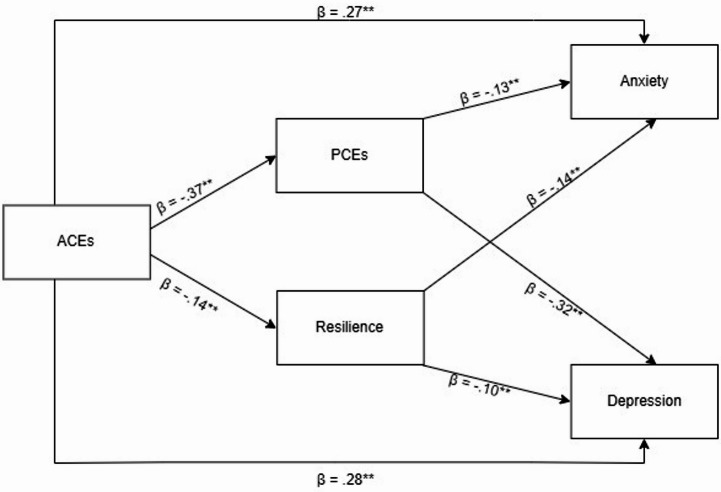
Parallel mediation model in adolescents

**Fig. 3:**
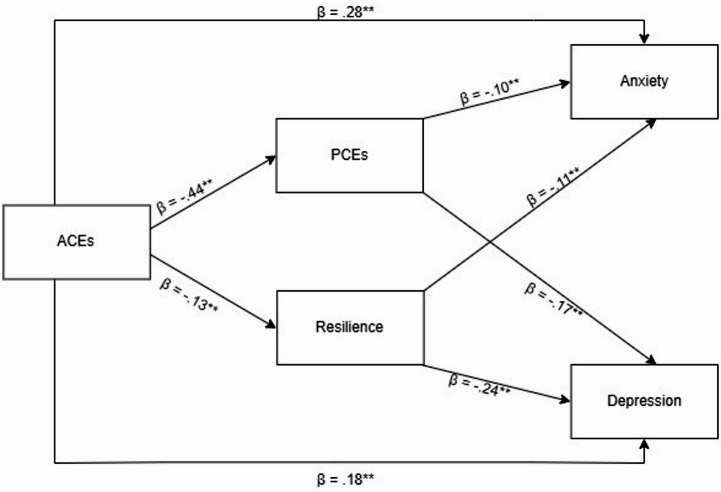
Parallel mediation model in young adults

After preliminary analyses, in the second stage, parallel mediation analyses were conducted to investigate the mediating roles of PCEs and resilience in the relationship between ACEs and anxiety and depression. Separate analyses were run for the adolescent, young adult, and adult sample groups included in the study (Figs. 1, 2 and 3; Tables 2 and 3). Mediation results for the adolescent group show that ACEs negatively and significantly predicted PCEs (β = − 0.37, *p*<.001) and resilience (β = − 0.14, *p*<.001), explaining 13% of the variance of PCEs and 2% of resilience. Furthermore, ACEs positively and significantly predicted anxiety (β = 0.27, *p*<.001) and depression (β = 0.28, *p*<.001), and ACEs together with PCEs and resilience explained 17% of the variance in anxiety and 30% of the variance in depression. Finally, the relationship between ACEs and anxiety and depression was mediated by PCEs (effect = 0.05–0.12, 95% CI [0.01, 0.16]) and resilience (effect = 0.02–0.01, 95% CI [0.001, 0.16]).

**Table 2:** Unstandardized coefficients for the mediation model

		Consequent					
		M_1_ (PCEs)					
Antecedent		Coeff.		*SE*		*t*	*p*
*X* (ACEs)		–1.20_a_		0.11		–10.44	< 0.001
		−1.24_Ya_		0.07		−16.08	< 0.001
		−1.22_Ad_		0.10		−11.96	< 0.001
Constant		25.36_a_		0.24		105.25	< 0.001
		25.72_Ya_		0.18		138.88	< 0.001
		26.31_Ad_		0.25		102.77	< 0.001
		_*a*_*R*^2^ = 0.13; *F* = 109.1; *p* <.001 _Ya_*R*^2^ = 0.19; *F* = 258.82; *p* <.001 _*Ad*_*R*^2^ = 0.21; *F* = 143.08; *p* <.001					
			*M*_*2*_ (Resilience)				
*X* (ACEs)			0.25_a_		0.06	−3.94	< 0.001
			0.18_Ya_		0.04	−4.40	< 0.001
			0.26_Ad_		0.05	−4.44	< 0.001
Constant			10.86_a_		0.13	81.24	< 0.001
			11.24_Ya_		0.09	114.80	< 0.001
			11.69		0.14	79.99	< 0.001
			_*a*_*R*^2^ = 0.02; *F* = 15.58; *p* <.001 _*Ya*_*R*^2^ = 0.01; *F* = 19.37; *p* <.001 _*Ad*_*R*^2^=0.03; *F* = 19.78; *p* <.001				
			*Y*_*1*_ (Anxiety)				
*X* (ACEs)			0.96_a_		0.12	7.47	< 0.001
			0.58_Ya_		0.08	6.68	< 0.001
			0.50_Ad_		0.10	4.71	< 0.001
*M*_*1*_ (PCEs)			–0.14_a_		0.04	−3.38	< 0.001
			−0.10_Ya_		0.03	−3.08	< 0.01
			0.09_Ad_		0.04	−2.23	< 0.05
*M*_*2*_ (Resilience)			−0.27_a_		0.07	−3.27	< 0.001
			−0.23_Ya_		0.06	−3.87	< 0.001
			0-.33_Ad_		0.07	−4.65	< 0.001
Constant			12.61_a_		1.13	11.12	< 0.001
			10.82_Ya_		0.94	11.46	< 0.001
			9.33_Ad_		1.26	7.35	< 0.001
			_*a*_*R*^2^ = 0.17; *F* = 46.34; *p* <.001 _*Ya*_*R*^2^ = 0.10; *F* = 41.57; *p* <.001 _*Ad*_*R*^2^ = 0.14; *F* = 28.84; *p* <.001				
			*Y*_*2*_ (Depression)				
*X(ACEs)*			0.91_a_		0.11	8.18	< 0.001
			0.50_Ya_		0.08	5.88	< 0.001
			0.36_Ad_		0.10	3.29	< 0.001
*M*_*1*_ *(PCEs)*			−0.32_a_		0.03	−9.01	< 0.001
			−0.17_Ya_		0.03	−5.46	< 0.001
			−0.20_Ya_		0.04	−4.97	< 0.001
*M*_*2*_ (Resilience)			−0.19_Ad_		0.06	−2.97	< 0.001
			−0.32_Ya_		0.05	−5.51	< 0.001
			−0.43_Ad_		0.07	−5.94	< 0.001
Constant			16.40_a_		0.98	16.61	< 0.001
			13.92_Ya_		0.91	15.20	< 0.001
			14.58_Ad_		1.29	11.24	< 0.001
			_*a*_*R*^2^ = 0.30; *F* = 97. 27; *p* <.001 _*Ya*_*R*^2^ = 0.14; *F* = 61. 40; *p* <.001 _*Ad*_*R*^2^ = 0.19; *F* = 43. 10; *p* <.001				

*SE* = standard error. Coeff = unstandardized coefficient. *X* = independent variable; *M* = mediator variable; *Y* = dependent variable. a= Adolescents, Ya= Young Adults, Ad= Adults

**Table 3:** ACEs indirectly affect anxiety and depression through PCEs and resilience

Sample groups	Indirect effects	Effect size	SE	95% Confidence Interval	
				Lower limit	Upper limit
Adolescents					
	ACEs→ PCEs→Anxiety	0.05	0.016	0.018	0.083
	ACEs→ PCEs→Depression	0.12	0.019	0.086	0.163
	ACEs→ Resilience→Anxiety	0.02	0.008	0.006	0.040
	ACEs→Resilience→Depression	0.01	0.007	0.003	0.031
Young Adults					
	ACEs→ PCEs→Anxiety	0.04	0.017	0.011	0.079
	ACEs→ PCEs→Depression	0.07	0.018	0.043	0.117
	ACEs→ Resilience→Anxiety	0.01	0.006	0.005	0.028
	ACEs→Resilience→Depression	0.02	0.007	0.009	0.037
Adults					
	ACEs→ PCEs→Anxiety	0.04	0.023	0.001	0.095
	ACEs→ PCEs→Depression	0.10	0.027	0.051	0.161
	ACEs→ Resilience→Anxiety	0.03	0.013	0.014	0.066
	ACEs→Resilience→Depression	0.04	0.015	0.019	0.081

Mediation results for the young adult group reveal that, as in the adolescent group, ACEs negatively and significantly predicted PCEs (β = − 0.37, *p*<.001) and resilience (β = − 0.13, *p*<.001), explaining 19% of the variance in PCEs and 1% in resilience. ACEs positively and significantly predicted anxiety (β = 0.28, *p*<.001) and depression (β = 0.18, *p*<.001), and together with ACEs, PCEs, and resilience, they explained 10% of the variance in anxiety and 14% of the variance in depression. Additionally, the relationship between ACEs and anxiety and depression was mediated by PCEs (effect = 0.07–0.04, 95% CI [0.01, 0.11]) and resilience (effect = 0.01–0.02, 95% CI [0.005, 0.09]).

**Fig. 4:**
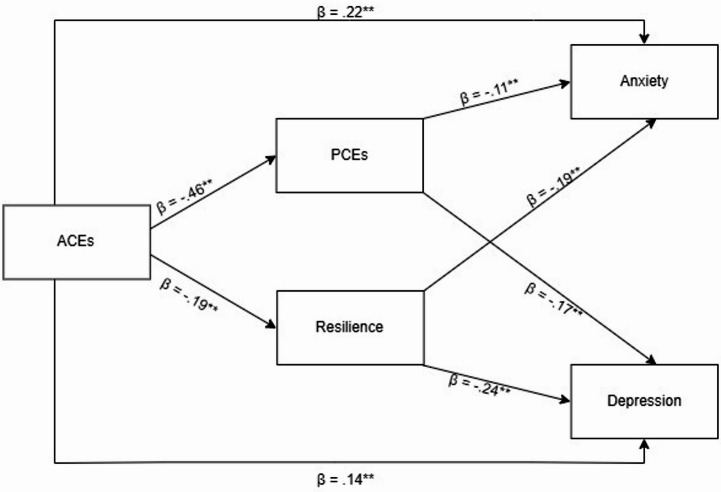
Parallel mediation model in adults

Finally, mediation results for the adult group show that, as in the adolescent and young adult groups, ACEs negatively and significantly predicted PCEs (β = − 0.46, *p*<.001) and resilience (β = − 0.19, *p*<.001), explaining 21% of the variance in PCEs and 3% in resilience. Furthermore, ACEs positively and significantly predicted anxiety (β = 0.22, *p*<.001) and depression (β = 0.14, *p*<.001), and ACEs together with PCEs and resilience explained 14% of the variance in anxiety and 19% of the variance in depression. Another mediation result found that the relationship between ACEs and anxiety and depression was mediated by PCEs (effect = 0.10–0.04, 95% CI [0.001, 0.16]) and resilience (effect = 0.03–0.04, 95% CI [0.01, 0.08]). All these analyses have found that the destructive effects of ACEs continue for a long time. On the other hand, PCEs and resilience can be considered strong parameters in buffering and neutralizing these adverse effects. (Figure 4.)

## Discussion

The current study aims to investigate the mediating roles of PCEs and resilience in the relationship between ACEs and anxiety and depression. The results of the study indicate that ACEs were negatively and significantly associated with PCEs and resilience and positively and significantly associated with anxiety and depression. These results suggest that having high ACEs may lead individuals to experience more anxiety and depression and lower resilience in adolescence, young adulthood, and adulthood. Additionally, the results obtained from the study reveal that individuals experience fewer PCEs. When these results are evaluated within the framework of the Developmental Psychopathology framework [59], it is reported that the destructive effects of ACEs last throughout life and that PCEs and resilience are two important elements in buffering these destructive effects. These results are also consistent with previous studies [9, 12, 14, 17, 74], and the existing literature suggests that adolescents and adults with high ACEs are more likely to experience depression and anxiety [8, 10]. In a study investigating ACEs in both childhood and adolescence, it was proven that ACEs positively predict anxiety and depression [75]. Similarly, ACEs increase adolescents’ anxiety and depression levels [65]. An inquiry on Chinese adults found that ACEs negatively and significantly predicted PCEs [40]. In the current literature, evidence pinpoints that exposure to high levels of ACEs is associated with lower resilience in adolescence [76] and adulthood [5, 77]. A study conducted with college students found that exposure to high ACEs was associated with lower psychological resilience [78].

The findings also support the current study’s (H_2_), “PCEs and resilience are negatively associated with anxiety and depression”. These results highlight that PCEs act as protective factors against mental health problems in childhood and adulthood, including anxiety and depression. Furthermore, the results of the study show that individuals with higher levels of resilience experience fewer symptoms of anxiety and depression. These findings are compatible with the previous studies [43, 52, 75]. In a study conducted on a large sample group, a negative and significant relationship was found between PCEs and anxiety and depression. Having higher PCEs makes individuals face fewer health problems. (Şanlı et al., 2024). A study of young adults found that PCEs reduce the risk of anxiety and depression (Chaundhary et al., 2025). Similarly, a study conducted on adolescents experiencing more PCEs reported lower anxiety and depression [65]. Additionally, a meta-analysis conducted by Hu et al., [79] indicates that resilience is negatively correlated with both anxiety and depression across various populations.

Finally, the relationship between ACEs and anxiety and depression was mediated by PCEs and resilience. This result shows that (H_3_) of the current study is confirmed. The results provide evidence that PCEs and resilience act as neutralizers against the effects of ACEs on different populations. On the other hand, no studies were found in the existing literature on the mediating roles of PCEs and resilience in the relationship between ACEs and anxiety and depression. Although limited, there are individual studies in the literature providing support for our research. In a study conducted on adolescents, PCEs were shown to moderate the relationship between ACEs and the risk of depression [65]. A high number of PCEs in young adulthood has been shown to both moderate the impact of ACEs and serve a protective role against their negative effects [14], and PCEs moderate the relationship between ACEs and anxiety, depression, and stress in adulthood. Furthermore, exposure to high levels of PCEs contributes to lower levels of psychological distress in adults [38]. Studies in the literature report that resilience holds a shielding role against ruinous situations. Resilience is a mitigating factor in the relationship between ACEs, childhood depression, depression, and poor mental health [74, 80, 81]. For instance, in a study conducted on university students, resilience mediated the relationship between ACEs and internet gaming disorder [82]. Similarly, resilience mediates the relationship between ACEs and depression in adolescents [57]. Moreover, positive factors such as resilience and school connectedness have been found to significantly moderate the relationship between ACEs and psychological distress, effectively mitigating the negative impact of ACEs [37]. Therefore, both PCEs and resilience can be regarded as two powerful protective resources in buffering against the harmful effects of ACEs.

### Theoretical and Practical Implications

The findings of the present study provide important theoretical and practical implications. From a theoretical standpoint, the results both support and extend the Developmental Psychopathology framework by demonstrating that the adverse effects of ACEs on anxiety and depression persist across adolescence, young adulthood, and adulthood. Moreover, the mediating roles of PCEs and psychological resilience underscore the dynamic and lifelong interplay between risk and protective factors. These findings contribute to the literature by highlighting that positive developmental processes can coexist with early adversity and continue to influence mental health outcomes well beyond childhood. From a practical perspective, our findings suggest the importance of strengthening PCEs and resilience across the lifespan to lessen the long-term impact of ACEs. Evidence-based programs such as school-based mentoring programs, social-emotional learning curricula, and resilience training models provide concrete ways to foster supportive relationships, emotional security, and adaptive coping skills. Grounded in positive psychology, these interventions have been shown to reduce symptoms of anxiety and depression among individuals exposed to childhood adversity, offering meaningful pathways for prevention and support. Furthermore, the findings emphasize the importance of policies and community-based programs that cultivate healthy and supportive environmental conditions during childhood. Such efforts not only buffer the detrimental effects of ACEs but also promote healthy development and overall well-being, even in the presence of early adverse experiences.

### Limitations and Future Directions

The ascendancy of this study is that it discusses the relationship between ACEs and anxiety and depression in adolescents, young adults, and adults through PCEs and resilience, further enriching the understanding of the adverse effects of ACEs. At the same time, this study also has some limitations. First, this study relies on cross-sectional data for discussion and has certain limitations in explaining the causal relationships among variables. Future longitudinal follow-up or experimental research may further enrich the explanation of these variables. This study relied on self-report surveys, which may involve social desirability bias and inaccurate self-perceptions. Future research should combine subjective and objective methods to strengthen validity and reduce bias. Finally, this study is based on scales conducted in the context of Turkish culture, and its interpretation in cross-cultural studies may need to be further strengthened. To overcome this limitation, researchers advise conducting comparative studies on individuals from different cultures and in the context of dissimilar regions.

## Conclusion

The present study further investigates the relationship between ACEs and anxiety and depression in adolescents, young adults, and adults, considering the mediating role of PCEs and resilience. The study results emphasize the necessity of strengthening PCEs and resilience at every stage of life to counteract the destructive effects of ACEs. Another important result of the present study asserts that the impact of ACEs on physical and mental health continues for many years. In every period of life, programs aimed at individuals to have more positive experiences in the context of positive psychology against the adverse consequences of ACEs emerge as an important parameter. Furthermore, PCEs and resilience have been shown to mitigate the effects of adversity, even in the presence of ACEs. Additionally, PCEs and resilience contribute to the enhancement of individuals’ healthy development and well-being. In this context, it is crucial to develop policies fostering healthy environmental conditions during childhood and to design supportive programs accordingly.

## Data Availability

The datasets generated during and/or analysed during the current study are available from the corresponding author upon reasonable request.

## References

[1] Bethell C, Jones J, Gombojav N, Linkenbach J, Sege R. Positive childhood experiences and adult mental and relational health in a statewide sample: associations across adverse childhood experiences levels. JAMA Pediatr. 2019;173(11):e193007-e193007. 10.1001/jamapediatrics.2019.3007. 31498386 10.1001/jamapediatrics.2019.3007 PMC6735495

[2] Felitti VJ, Anda RF, Nordenberg D, Williamson DF, Spitz AM, Edwards V, Marks JS. Relationship of childhood abuse and household dysfunction to many of the leading causes of death in adults: The Adverse Childhood Experiences (ACE) Study. American journal of preventive medicine, 1998;14(4):245–58. 10.1016/S0749-3797(98)00017-8. 10.1016/s0749-3797(98)00017-8 9635069

[3] Anda RF, Butchart A, Felitti VJ, Brown DW. Building a framework for global surveillance of the public health implications of adverse childhood experiences. Am J Prev Med. 2010;39(1):93–8. 10.1016/j.amepre.2010.03.015. 20547282 10.1016/j.amepre.2010.03.015

[4] Cicchetti D, Toth SL. The past achievements and future promises of developmental psychopathology: The coming of age of a discipline. J Child Psychol Psychiatry. 2009;50(1–2):16–25. 10.1111/j.1469-7610.2008.01979.x. 19175810 10.1111/j.1469-7610.2008.01979.x PMC3531893

[5] Hughes K, Bellis MA, Hardcastle KA, Sethi D, Butchart A, Mikton C, Dunne MP. The effect of multiple adverse childhood experiences on health: a systematic review and meta-analysis. Lancet Public Health. 2017;2(8):e356–66. 10.1016/S2468-2667(17)30118-4. 29253477 10.1016/S2468-2667(17)30118-4

[6] Kallapiran K, Suetani S, Cobham V, Eapen V, Scott J. Impact of Positive Childhood Experiences (PCEs): A Systematic Review of Longitudinal Studies. Child Psychiatry Hum Dev. 2025;1–16. 10.1007/s10578-024-01807-x. 10.1007/s10578-024-01807-x PMC13615033 39760826

[7] Bhutta ZA, Bhavnani S, Betancourt TS, Tomlinson M, Patel V. Adverse childhood experiences and lifelong health. Nat Med. 2023;29(7):1639–48. 37464047 10.1038/s41591-023-02426-0

[8] Bellis MA, Hughes K, Ford K, Ramos Rodriguez G, Sethi D, Passmore J. Life course health consequences and associated annual costs of adverse childhood experiences across Europe and North America: a systematic review and meta-analysis. Lancet Public Health. 2019;4:e517–28. 10.1016/S2468-2667(19)30145-8. 31492648 10.1016/S2468-2667(19)30145-8 PMC7098477

[9] Iniguez KC, Stankowski RV. Adverse childhood experiences and health in adulthood in a rural population-based sample. Clin Med Res. 2016;14:126–37. 10.3121/cmr.2016.1306. 27503793 10.3121/cmr.2016.1306 PMC5302459

[10] Jia Z, Wen X, Chen F, Zhu H, Li C, Lin Y, et al. Cumulative exposure to adverse childhood experience: depressive symptoms, suicide intensions and suicide plans among senior high school students in Nanchang City of China. Int J Environ Res Public Health. 2020;17:4718. 10.3390/ijerph17134718. 32630073 10.3390/ijerph17134718 PMC7369761

[11] Lin WH, Chiao C. Adverse childhood experience and young adult’s problematic internet use: the role of hostility and loneliness. Child Abuse Negl. 2024;149:106624. 10.1016/j.chiabu.2023.106624. 38227984 10.1016/j.chiabu.2023.106624

[12] Mersky JP, Topitzes J, Reynolds AJ. Impacts of adverse childhood experiences on health, mental health, and substance use in early adulthood: A cohort study of an urban, minority sample in the US. Child Abuse Negl. 2013;37(11):917–25. 10.1016/j.chiabu.2013.07.011. 23978575 10.1016/j.chiabu.2013.07.011 PMC4090696

[13] Petruccelli K, Davis J, Berman T. Adverse childhood experiences and associated health outcomes: A systematic review and meta-analysis. Child Abuse Negl. 2019;97:104127. 10.1016/j.chiabu.2019.104127. 31454589 10.1016/j.chiabu.2019.104127

[14] Chaudhary V, Walia GK, Devi NK, Saraswathy KN. Prevalence and predictors of positive childhood experiences and their relationship with adverse childhood experiences among young adults in Delhi-NCR, India. Int J Soc Psychiatry. 2025;00207640241310188. 10.1177/00207640241310188. 10.1177/00207640241310188 39791918

[15] Lee AH, Mahurkar-Joshi S, Naliboff B, Gupta A, Labus J, Tillisch K, Chang L. Role of sex, anxiety, and resilience in the association between adverse childhood experiences and irritable bowel syndrome. Clin Gastroenterol Hepatol. 2025;23(1):154–62. 10.1016/j.cgh.2024.05.041. 38878847 10.1016/j.cgh.2024.05.041 PMC11648812

[16] Van Doorn G, Dye J, Teese R. Adverse and positive childhood experiences and their associations with dark personality traits. J Res Pers. 2025;104583. 10.1016/j.jrp.2025.104583.

[17] Zhang X, Li C, Ma W. The direct and indirect effects of adverse childhood experiences on depressive symptoms and self-esteem of children: Does gender make a difference? Int J Ment Health Addict. 2024;22(1):254–78. 10.1007/s11469-022-00871-5.

[18] Cunha O, Sousa M, Pereira B, Pinheiro M, Machado AB, Caridade S, et al. Positive childhood experiences and adult outcomes: a systematic review. Trauma Violence Abuse. 2024;15248380241299434. 10.1177/15248380241299434. 10.1177/15248380241299434 PMC12569125 39614772

[19] Ramaj K, Eisner M. Adverse childhood experiences, intimate partner violence, and mental well-being among mothers of toddlers in Tirana, Albania: a cross-sectional mediation analysis. Violence Against Women. 2025;31(1):206–23. 37774772 10.1177/10778012231203659 PMC11610198

[20] Chi X, Jiang W, Guo T, Hall DL, Luberto CM, Zou L. Relationship between adverse childhood experiences and anxiety symptoms among Chinese adolescents: The role of self-compassion and social support. Curr Psychol. 2023;42:12822–34. 10.1007/s12144-021-02534-5. 10.1007/s12144-021-02534-5 PMC8741560 35035184

[21] Crandall A, Miller JR, Cheung A, Novilla LK, Glade R, Novilla MLB, et al. ACEs and counter-ACEs: How positive and negative childhood experiences influence adult health. Child Abuse Negl. 2019;96:104089. 10.1016/j.chiabu.2019.104089. 31362100 10.1016/j.chiabu.2019.104089

[22] Lee HY, Kim I, Nam S, Jeong J. Adverse childhood experiences and the associations with depression and anxiety in adolescents. Child Youth Serv Rev. 2020;111:104850.

[23] Dosanjh LH, Lauby S, Fuentes J, Castro Y, Conway F, Champagne FA, Goosby B. Five Hypothesized Biological Mechanisms Linking Adverse Childhood Experiences with Anxiety, Depression, and PTSD: A Scoping Review. Neurosci Biobehavioral Reviews. 2025;106062. 10.1016/j.neubiorev.2025.106062.10.1016/j.neubiorev.2025.10606239952339

[24] American Psychiatric Association. Diagnostic and statistical manual of mental disorders. 5th ed. Arlington, VA: American Psychiatric Association; 2013.

[25] Daniali H, Martinussen M, Flaten MA. A global meta-analysis of depression, anxiety, and stress before and during COVID-19. Health Psychol. 2023;42(2):124. 10.1037/hea0001259. 36802363 10.1037/hea0001259

[26] World Health Organization. /2017.2). Depression and other common mental disorders: global health estimates. WHO/MSD/MER: World Health Organization; 2017.

[27] Song H, Zhao Y, Hu C, Zhao C, Wang X, Xiao Z. Relationships among anxiety, depression, and health-related quality of life in adult epilepsy: a network analysis. Epilepsy Behav. 2024;154:109748. 38640553 10.1016/j.yebeh.2024.109748

[28] Zhang C, Ye M, Fu Y, Yang M, Luo F, Yuan J, et al. The psychological impact of the COVID-19 pandemic on teenagers in China. J Adolesc Health. 2020;67(6):747–55. 10.1016/j.jadohealth.2020.08.026. 33041204 10.1016/j.jadohealth.2020.08.026 PMC7543885

[29] Elmore AL, Crouch E. The association of adverse childhood experiences with anxiety and depression for children and youth, 8 to 17 years of age. Acad Pediatr. 2020;20(5):600–8. 10.1016/j.acap.2020.02.012. 32092442 10.1016/j.acap.2020.02.012 PMC7340577

[30] Watt T, Ceballos N, Kim S, Pan X, Sharma S. The unique nature of depression and anxiety among college students with adverse childhood experiences. J Child Adolesc Trauma. 2020;13:163–72. 10.1007/s40653-019-00270-4. 32549928 10.1007/s40653-019-00270-4 PMC7289944

[31] Whiteford HA, Degenhardt L, Rehm J, Baxter AJ, Ferrari AJ, Erskine HE, et al. Global burden of disease attributable to mental and substance use disorders: findings from the Global Burden of Disease Study 2010. Lancet. 2013;382(9904):1575–86. 10.1016/S0140-6736(13)61611-6. 23993280 10.1016/S0140-6736(13)61611-6

[32] Çiçek İ, Çeri V. Olumlu çocukluk yaşantıları ölçeği: Türkçe geçerlik ve güvenirlik çalışması. Humanistic Perspective. 2021;3(3):643–59. 10.47793/hp.980149.

[33] Baglivio MT, Wolff KT. Positive childhood experiences (PCE): cumulative resiliency in the face of adverse childhood experiences. Youth Violence Juv Justice. 2021;19(2):139–62. 10.1177/1541204020972487.

[34] Şanli ME, Çiçek İ, Yıldırım M, Çeri V. Positive childhood experiences as predictors of anxiety and depression in a large sample from Turkey. Acta Psychol. 2024;243:104170. 10.1016/j.actpsy.2024.104170. 10.1016/j.actpsy.2024.104170 38301406

[35] Çeri V, Çicek I. Psychological well-being, depression and stress during COVID-19 pandemic in Turkey: a comparative study of healthcare professionals and non-healthcare professionals. Psychol Health Med. 2021;26(1):85–97. 10.1080/13548506.2020.1859566. 33320723 10.1080/13548506.2020.1859566

[36] Hou H, Zhang C, Tang J, Wang J, Xu J, Zhou Q, et al. Childhood experiences and psychological distress: can benevolent childhood experiences counteract the negative effects of adverse childhood experiences? Front Psychol. 2022;13:800871. 10.3389/fpsyg.2022.800871. 35282200 10.3389/fpsyg.2022.800871 PMC8914177

[37] Clements-Nolle K, Waddington R. Adverse childhood experiences and psychological distress in juvenile offenders: The protective influence of resilience and youth assets. J Adolesc Health. 2019;64(1):49–55. 10.1016/j.jadohealth.2018.09.025. 30579436 10.1016/j.jadohealth.2018.09.025

[38] Nahar K, Mousum S, Salwa M, Fatema K, Chowdhury T, Tasnim A, et al. Childhood echoes: how benevolent and adverse childhood experiences shape adult mental well-being. Child Abuse Negl. 2025. 10.1016/j.chiabu.2025.107308. 39955962 10.1016/j.chiabu.2025.107308

[39] Crandall A, Broadbent E, Stanfill M, Magnusson BM, Novilla MLB, Hanson CL, et al. The influence of adverse and advantageous childhood experiences during adolescence on young adult health. Child Abuse Negl. 2020;108:104644. 32795716 10.1016/j.chiabu.2020.104644

[40] Geng F, Zou J, Liang Y, Zhan N, Li S, Wang J. Associations of positive and adverse childhood experiences and adulthood insomnia in a community sample of Chinese adults. Sleep Med. 2021;80:46–51. 10.1016/j.sleep.2021.01.022. 33550174 10.1016/j.sleep.2021.01.022

[41] Kocatürk M, Çiçek İ. Relationship between positive childhood experiences and psychological resilience in university students: the mediating role of self-esteem. J Psychologists Counsellors Schools. 2023;33(1):78–89. 10.1017/jgc.2021.16.

[42] Ünsal F, Korkmaz Z, Çiçek İ, Abdullah Alshehri N, Mohammed Abdullah Alkhulayfi A, Yıldırım M. Mediating roles of self-esteem and positive childhood experiences in the relationship between problematic social media use and loneliness. Psicol Reflex Crit. 2025;38(1):28. 10.1186/s41155-025-00364-z. 41082046 10.1186/s41155-025-00364-z PMC12518197

[43] Han D, Dieujuste N, Doom JR, Narayan AJ. A systematic review of positive childhood experiences and adult outcomes: Promotive and protective processes for resilience in the context of childhood adversity. Child Abuse Negl. 2023;144:106346. 10.1016/j.chiabu.2023.106346. 37473619 10.1016/j.chiabu.2023.106346 PMC10528145

[44] Çiçek İ, Korkmaz Z, Ünsal F, Shalal Alanazi Z, Gómez-Salgado J, Yildirim M. The effect of secure attachment on family relationships and peer bullying in adolescents: the mediating role of positive childhood experiences. Front Psychol. 2025;16:1700648. 10.3389/fpsyg.2025.1700648. 41312272 10.3389/fpsyg.2025.1700648 PMC12646994

[45] Hinojosa MS, Hinojosa R. Positive and adverse childhood experiences and mental health outcomes of children. Child Abuse Negl. 2024;149:106603. 10.1016/j.chiabu.2023.106603. 38141478 10.1016/j.chiabu.2023.106603

[46] Masten AS. Ordinary magic: resilience processes in development. Am Psychol. 2001;56(3):227–38. 10.1037/0003-066X.56.3.227. 11315249 10.1037//0003-066x.56.3.227

[47] Ryff CD, Singer B. Flourishing under fire: resilience as a prototype of challenged thriving. In: Keyes CLM, Haidt J, editors. Flourishing: positive psychology and the life well-lived. American Psychological Association; 2003. p. 15–36. 10.1037/10594-001

[48] Smith BW, Dalen J, Wiggins K, Tooley E, Christopher P, Bernard J. The brief resilience scale: assessing the ability to bounce back. Int J Behav Med. 2008;15:194–200. 10.1080/10705500802222972. 18696313 10.1080/10705500802222972

[49] Bonanno GA. Loss, trauma, and human resilience: have we underestimated the human capacity to thrive after extremely aversive events? Psychol Trauma Theory Res Pract Policy. 2008;S(1):101–13. 10.1037/1942-9681.S.1.101. 10.1037/0003-066X.59.1.20 14736317

[50] Yıldırım M, Çiçek I. (2022). Fear of COVID-19 and smartphone addiction among Turkish adolescents: Mitigating role of resilience. Family J, 10664807221139510.

[51] Stratta P, Capanna C, Patriarca S, de Cataldo S, Bonanni RL, Riccardi I, et al. Resilience in adolescence: gender differences two years after the earthquake of L’Aquila. Pers Individ Differ. 2013;54(3):327–31. 10.1016/j.paid.2012.09.016.

[52] Leys C, Kotsou I, Shankland R, Firmin M, Péneau S, Fossion P. Resilience predicts lower anxiety and depression and greater recovery after a vicarious trauma. Int J Environ Res Public Health. 2021;18(23):12608. 34886346 10.3390/ijerph182312608 PMC8656954

[53] Watters ER, Aloe AM, Wojciak AS. Examining the associations between childhood trauma, resilience, and depression: a multivariate meta-analysis. Trauma Violence Abuse. 2023;24(1):231–44. 10.1177/15248380211029397. 34313169 10.1177/15248380211029397

[54] Kim-Cohen J. Resilience and developmental psychopathology. Child Adolesc Psychiatr Clin N Am. 2007;16(2):271–83. 10.1016/j.chc.2006.11.003. 17349508 10.1016/j.chc.2006.11.003

[55] Koelmel E, Hughes AJ, Alschuler KN, Ehde DM. Resilience mediates the longitudinal relationships between social support and mental health outcomes in multiple sclerosis. Arch Phys Med Rehabil. 2017;98(6):1139–48. 10.1016/j.apmr.2016.09.127. 27789238 10.1016/j.apmr.2016.09.127

[56] Yıldırım M, Arslan G. Exploring the associations between resilience, dispositional hope, preventive behaviours, subjective well-being, and psychological health among adults during early stage of COVID-19. Curr Psychol. 2022;41(8):5712–22. 10.1007/s12144-020-01177-2. 33223782 10.1007/s12144-020-01177-2 PMC7666616

[57] Shahidi M, Ungar M, Wedyaswari M, Shojaee M. The role of resilience as a mediating factor between adverse childhood experience and mental health in adolescents receiving child welfare services in Nova Scotia. Child Adolesc Soc Work J. 2024. 10.1007/s10560-024-00979-8.

[58] Willis MC, Jeffries J, Barrett AR, Swearer SM. The impact of positive and adverse childhood experiences on social connectedness in young adults. J Exp Child Psychol. 2024;247:106033. . 10.1016/j.jecp.2024.106033 39137506 10.1016/j.jecp.2024.106033

[59] Cicchetti D, Rogosch FA. A developmental psychopathology perspective on adolescence. J Consult Clin Psychol. 2002;70(1):6. 10.1037/0022-006X.70.1.6. 11860057 10.1037//0022-006x.70.1.6

[60] Masten AS, Cicchetti D. Resilience in development: progress and transformation. Dev psychopathology: Risk Resil intervention. 2016;4:271–333. 10.1002/9781119125556.devpsy406.

[61] Chartier MJ, Walker JR, Naimark B. Health risk behaviors and mental health problems as mediators of the relationship between childhood abuse and adult health. Am J Public Health. 2009;99(5):847–54. 10.2105/AJPH.2007.122408. 18703446 10.2105/AJPH.2007.122408 PMC2667838

[62] Chen SS, He Y, Xie GD, Chen LR, Zhang TT, Yuan MY, et al. Relationships among adverse childhood experience patterns, psychological resilience, self-esteem and depressive symptoms in Chinese adolescents: A serial multiple mediation model. Prev Med. 2022;154:106902. 10.1016/j.ypmed.2021.106902. 34863811 10.1016/j.ypmed.2021.106902

[63] Kascakova N, Furstova J, Hasto J, Tavel P. Associations of multiple adverse childhood experiences, attachment insecurity and loneliness with physical and mental health difficulties in a representative Slovak sample. Prev Med Rep. 2025;102982. 10.1016/j.pmedr.2025.102982. 10.1016/j.pmedr.2025.102982 PMC11788736 39901935

[64] Crouch E, Andersen TS, Smith HP. Adverse childhood experiences and positive childhood experiences among United States military children. Mil Med. 2024;189(5–6):e1072–9. 10.1093/milmed/usad416. 37897695 10.1093/milmed/usad416

[65] Qu G, Ma S, Liu H, Han T, Zhang H, Ding X, Sun Y. Positive childhood experiences can moderate the impact of adverse childhood experiences on adolescent depression and anxiety: Results from a cross-sectional survey. Child Abuse Negl. 2022;125:105511. 10.1016/j.chiabu.2022.105511. 35078091 10.1016/j.chiabu.2022.105511

[66] Merino M, Privado J. Funcionamiento psicológico positivo: evidencia para un nuevo constructo ysú medición. An Psicol. 2015;31(1):45–54. 10.6018/analesps.31.1.171081.

[67] Green ZA, Çiçek İ, Yıldırım M. The relationship between social support and uncertainty of COVID-19: the mediating roles of resilience and academic self-efficacy. Psihologija. 2024;00:2–2. 10.2298/PSI220903002G.

[68] Lovibond PF, Lovibond SH. The structure of negative emotional states: comparison of the Depression Anxiety Stress Scales (DASS) with the Beck Depression and Anxiety Inventories. Behav Res Ther. 1995;33(3):335–43. 10.1016/0005-7967(94)00075-U. 7726811 10.1016/0005-7967(94)00075-u

[69] Henry JD, Crawford JR. The short-form version of the Depression Anxiety Stress Scales (DASS-21): construct validity and normative data in a large non-clinical sample. Br J Clin Psychol. 2005;44(2):227–39. 10.1348/014466505X29657. 16004657 10.1348/014466505X29657

[70] Yılmaz Ö, Boz H, Arslan A. Depresyon anksiyete stres ölçeğinin(dass 21) Türkçe kısa formunun geçerlilik-güvenilirlik çalışması. Finans Ekonomi ve Sosyal Araştırmalar Dergisi. 2017;2(2):78–91.

[71] Hayes AF. Introduction to mediation, moderation, and conditional process analysis: A regression-based approach. Guilford; 2017.

[72] Kline RB. Principles and practice of structural equation modeling. Guilford; 2023.

[73] Kline P. A handbook of test construction (psychology revivals): Introduction to psychometric design. New York: Routledge; 2015.

[74] Elmore AL, Crouch E, Chowdhury MAK. The interaction of adverse childhood experiences and resiliency on the outcome of depression among children and youth, 8–17 year olds. Child Abuse Negl. 2020;107:104616. 32645587 10.1016/j.chiabu.2020.104616 PMC7494539

[75] Zhu S, Liu Y, Ying J, Jiang D, Xiao W, Zhou J, Song P. Timing of adverse childhood experiences and depressive, anxiety, comorbid symptoms among Chinese female nurses: A life course perspective. Child Abuse Negl. 2025;161:107254. 10.1016/j.chiabu.2025.107254. 39862645 10.1016/j.chiabu.2025.107254

[76] Soleimanpour S, Geierstanger S, Brindis CD. Adverse childhood experiences and resilience: addressing the unique needs of adolescents. Acad Pediatr. 2017;17(7):S108-14. 10.1016/j.acap.2017.01.008. 28865641 10.1016/j.acap.2017.01.008

[77] Haczkewicz KM, Shahid S, Finnegan HA, Moninn C, Cameron CD, Gallant NL. Adverse childhood experiences (ACEs), resilience, and outcomes in older adulthood: A scoping review. Child Abuse Negl. 2024;106864. 10.1016/j.chiabu.2024.106864. 10.1016/j.chiabu.2024.106864 38926006

[78] Canatan SD, Arifoğlu B, Yatmaz G. Adverse childhood events of individuals and its relationship with resilience. Arch Psychiatr Nurs. 2024;51:114–9. 10.1016/j.apnu.2024.05.003. 39034066 10.1016/j.apnu.2024.05.003

[79] Hu T, Zhang D, Wang J. A meta-analysis of the trait resilience and mental health. Pers Individ Differ. 2015;76:18–27. 10.1016/j.paid.2014.11.039.

[80] Kelifa MO, Yang Y, Herbert C, He Q, Wang P. Psychological resilience and current stressful events as potential mediators between adverse childhood experiences and depression among college students in Eritrea. Child Abuse Negl. 2020;106:104480. 10.1016/j.chiabu.2020.104480. 32470689 10.1016/j.chiabu.2020.104480

[81] Liu M, Mejia-Lancheros C, Lachaud J, Nisenbaum R, Stergiopoulos V, Hwang SW. Resilience and adverse childhood experiences: associations with poor mental health among homeless adults. Am J Prev Med. 2020;58(6):807–16. 10.1016/j.amepre.2019.12.017. 32147372 10.1016/j.amepre.2019.12.017

[82] Ma J, Yang B, Wang S, Yao Y, Wu C, Li M, Dong GH. Adverse childhood experiences predict internet gaming disorder in university students: the mediating role of resilience. Curr Opin Psychiatry. 2024;37(1):29–37. 10.1097/YCO.0000000000000910. 37972967 10.1097/YCO.0000000000000910

